# Characterization of Solvent-Treated PEDOT:PSS Thin Films with Enhanced Conductivities

**DOI:** 10.3390/polym11010134

**Published:** 2019-01-14

**Authors:** Syang-Peng Rwei, Yi-Huan Lee, Jia-Wei Shiu, Ragu Sasikumar, Uin-Ting Shyr

**Affiliations:** 1 Institute of Organic and Polymeric Materials, National Taipei University of Technology, Taipei 10608, Taiwan; yihuanlee@mail.ntut.edu.tw (Y.-H.L.); hahaluckybowen@gmail.com (J.-W.S.); alexilaiho0422@gmail.com (U.-T.S.); 2 Research and Development Center for Smart Textile Technology, National Taipei University of Technology, Taipei 10608, Taiwan; sasi.che2011@gmail.com

**Keywords:** PEDOT:PSS thin film, conducting polymer, electrical conductivity, X-ray diffraction

## Abstract

The conducting polymer of poly(3,4-ethylenedioxythiophene):poly(4-styrenesulfonate) (PEDOT:PSS) is one of the most important and intensively investigated organic conducting materials. The PEDOT:PSS water dispersions with various concentrations of poly (4-styrenesulfonic acid) solution (PSSAS) were synthesized by 3,4-ethylenedioxythiophene (EDOT) in the presence of water. The fabrication of the solvent-treated PEDOT:PSS films through spin coating and solvent treatment processes was achieved with a solvent of pure water mixed with acetone (or MeOH, EtOH) in a ratio of 50:50. Moreover, both the organic solvent and water have synergetic effects while the PSS and PEDOT-attached PSS segments will form a coil-like and a linear (or extended-coil) structure, respectively. That may induce a stacking of the linear and planar PEDOT-attached PSS segments, which favors the formation of a crystalline phase. Finally, the maximum electrical conductivity of the PEDOT:PSS thin films with solvent treatment was investigated by means of X-ray diffraction (XRD) patterns and scanning electron microscope (SEM) images. Furthermore, we aimed to explain the synergetic effects of phase separation of the PEDOT:PSS thin films by both the organic solvent and water.

## 1. Introduction

Currently, organic conducting polymers have attracted interest in electronics due to their light weight, low cost and solution processible manufacturability. Moreover, polythiophene derivatives are one of the most successful conducting polymers due to their environmental stability, thermal stability, excellent film-forming ability and transparency [1,2,3]. However, polythiophene derivatives, such as poly(3,4-ethylene dioxythiophene) (PEDOT), are difficult to solvate in any solvent when in the conductive state. However, excess poly(4-styrenesulfonate) (PSS) that can be dispersed in water with good stability has been added in order to solve this problem. Furthermore, the conducting polymer of poly(3,4-ethylenedioxythiophene):poly(4-styrenesulfonate) (PEDOT:PSS) is one of the most important and intensively investigated organic conducting materials. This has the advantages of excellent film-forming performance, flexibility, wet-process ability, transparency, thermal stability and high conductivity for applications, such as wearable electronics, flexible displays, touch panels, solar cells, smart sensors, antistatic coatings, solid electrolytic capacitors and organic LEDs [4,5,6,7,8,9,10,11]. In addition, PEDOT:PSS thin films can enhance high conductivity through different film-forming methods, such as an addition of organic solvents (e.g., dimethyl sulfoxide (DMSO), ethylene glycol (EG)) and ionic liquid, spin coating, drop casting and diluting filtration. Moreover, the electrical conductivity of optimized PEDOT:PSS films can reach up to 1000 and 4000 S/cm by polar-solvent vapor annealing methods and post-treatment with sulfuric acid, respectively [12,13,14,15,16,17,18]. Above all, conductivity can be enhanced by the addition of a polar organic solvent with a high boiling point, such as DMSO or EG, but not by a pure organic solvent with a low boiling point, such as acetone, methanol (MeOH), ethanol (EtOH), iso-propyl alcohol (IPA), acetonitrile (ACN) or tetrahydrofuran (THF). Thus, the electrical conductivity of PEDOT:PSS thin films that are treated by a pure organic solvent with a low boiling point is between 0.4 and 14.8 S/m. On the other hand, the conductivity enhancement of the PEDOT:PSS thin films can be achieved with a co-solvent of water mixed with an organic solvent, such as acetone, MeOH, EtOH, IPA, ACN or THF. Thus, the electrical conductivity of PEDOT:PSS films that are treated by a co-solvent is between 40.9 and 78.9 S/m. The enhancement of conductivity was attributed to the preferential solvation of the PEDOT and PSS chains with the co-solvents. Furthermore, the electrical conductivity can be as high as 700 S/cm at the optimum composition ratio between the PEDOT and PSS [19,20].

In this study, we synthesized conductive PEDOT:PSS dispersions with various PSS ratios through oxidative polymerization. The significant enhancement of the conductivity of PEDOT:PSS thin films through solvent treatments of the water mixed with an organic solvent, such as acetone, MeOH and EtOH, are analyzed by means of X-ray diffraction (XRD) patterns and scanning electron microscope (SEM) images in order to explain the synergetic effects of phase separation of the PEDOT:PSS thin films by both the organic solvent and water.

## 2. Experimental

### 2.1. Materials

Quantities of 3,4-ethylenedioxythiophene (EDOT, 97%), poly (4-styrenesulfonic acid) solution (PSSAS, *M*_w_:75,000), Amberlyst^®^ 15 hydrogen as a cation exchange resin and Amberlyst^®^ A26 hydroxide as an anion exchange resin were purchased from Sigma-Aldrich, Inc., Taipei, Taiwan. Iron(II) sulfate heptahydrate (FeSO_4_·7H_2_O) as a catalyst, sodium persulfate (Na_2_S_2_O_8_) as an oxidation agent and hydrochloric acid (HCl, 37%) were purchased from Showa Chemical Industry Co., Ltd., Tokyo, Japan. Commercial PEDOT:PSS (ORGACON ICP1050) was purchased from Agfa Taiwan Co., Ltd., Taipei, Taiwan. All other chemicals, solvents and reagents used were of analytical grade. Deionized water (DI water) was used in all experiments.

### 2.2. Fabrication of PEDOT:PSS Dispersions

**Scheme 1:**
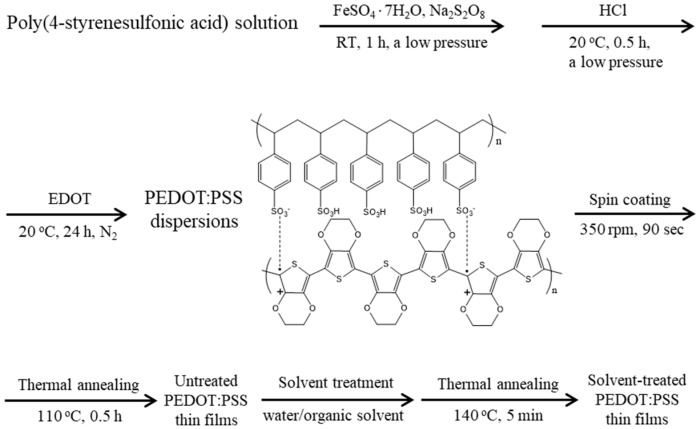
Processes of the poly(3,4-ethylenedioxythiophene):poly(4-styrenesulfonate) (PEDOT:PSS) water dispersions, untreated PEDOT:PSS thin films and the solvent-treated PEDOT:PSS thin films.

The PEDOT:PSS dispersions with various concentrations of PSSAS (2.4, 4.8 and 6.2%) were synthesized by EDOT in the presence of 200 mL of water. First, PSSAS were mixed in 200 mL of water containing 0.98 wt% of Na_2_S_2_O_8_ as an oxidation agent and 0.2 wt% of FeSO_4_·7H_2_O as a catalyst under low pressure conditions with a motor pump at room temperature (RT) for 1 h. After this, 305 μL of HCl was mixed in water under low pressure conditions with a motor pump at 20 °C for 30 min before 751 μL of EDOT was added to water under a nitrogen atmosphere at 20 °C for 24 h. After polymerization, the resulting sodium, iron and sulfate ions were removed using the cation exchange (Amberlyst^®^ 15) and anion exchange (Amberlyst^®^ A26) resins. The processes of the PEDOT:PSS water dispersions and the solvent-treated PEDOT:PSS thin films are shown in Scheme 1.

### 2.3. Fabrication of Untreated PEDOT:PSS Thin Films

The untreated PEDOT:PSS thin films were fabricated by spin coating. PEDOT:PSS dispersions were dropped onto a glass substrate at 350 rpm for 90 s. Thermal annealing was conducted under an air atmosphere at 110 °C for 30 min to obtain untreated PEDOT:PSS thin films (film A, B and C, for 2.4, 4.8 and 6.2% PSSAS, respectively) (Scheme 1).

### 2.4. Fabrication of Solvent-Treated PEDOT:PSS Thin Films

Pure water was mixed with acetone (MeOH or EtOH) in a ratio of 50:50 as the solvent for treating the PEDOT:PSS thin films. The solvent was dropped onto a untreated PEDOT:PSS thin film (A, B or C) before thermal annealing was conducted under an air atmosphere at 140 °C for 5 min to obtain solvent-treated PEDOT:PSS thin films (Scheme 1).

### 2.5. Measurements

X-ray diffraction (XRD) patterns were measured by Malvern Panalytical, Empyrean. Surface morphology was investigated by means of a scanning electron microscope (SEM, JEOL/JSM-7610F). Electrical conductivity of the PEDOT:PSS thin films was measured by a normal four-point method (Loresta-GP MCP-T600, Mitsubishi Chemical Analytech Co., Ltd., Kanagawa, Japan).

## 3. Results and Discussion

### 3.1. XRD Measurements of the PEDOT:PSS Thin Films

**Figure 1:**
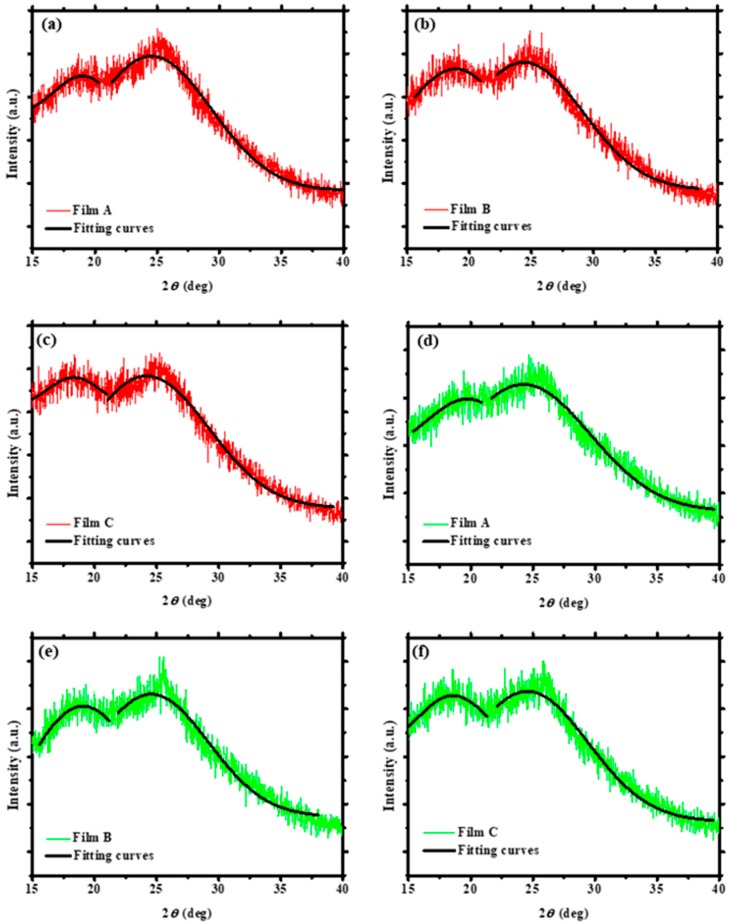

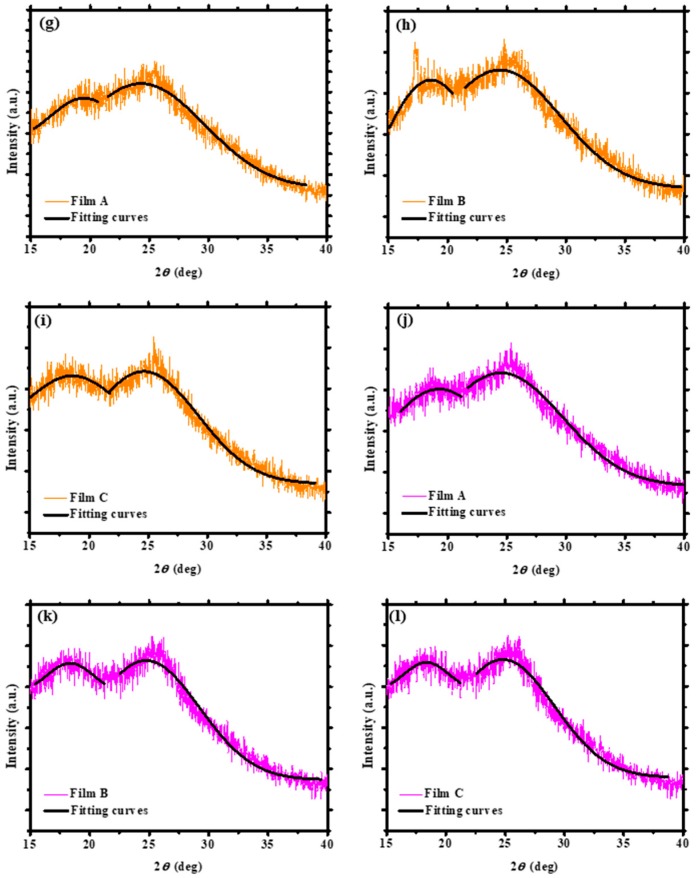
X-ray diffraction (XRD) spectra and fitting curves of the PEDOT:PSS thin film A (2.4% PSSAS), B (4.8% PSSAS) and C (6.2% PSSAS) without solvent treatments (**a**–**c**) and with solvent treatments of water/acetone (**d**–**f**), water/methanol (**g**–**i**) and water/ethanol (**j**–**l**).

**Figure 2:**
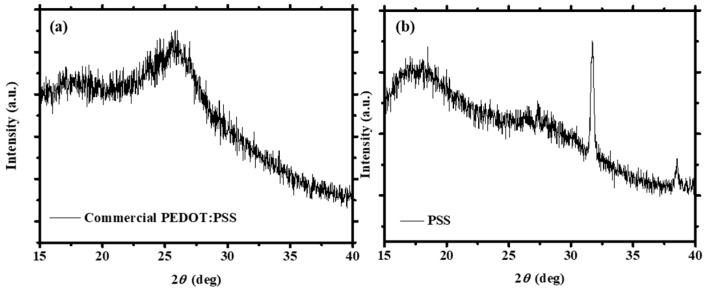
XRD spectra for the (**a**) commercial PEDOT:PSS and (**b**) PSSAS powder.

**Scheme 2:**
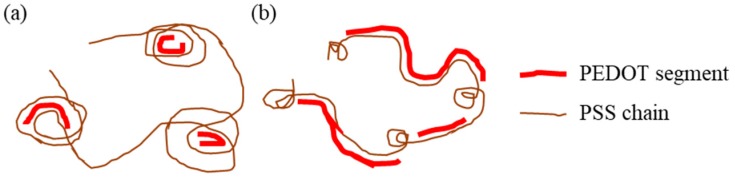
Conformations of PEDOT:PSS: (**a**) untreated PEDOT:PSS and (**b**) solvent-treated PEDOT:PSS.

The X-ray patterns and fitting curves of the PEDOT:PSS thin films (film A, B and C for 2.4, 4.8 and 6.2% PSSAS, respectively), which were either untreated and prepared by spin coating and thermal annealing or solvent-treated and prepared by the solvent treatment and thermal annealing (as shown in Scheme 1), are shown in Figure 1. According to Figure 1, all of the peaks from the PEDOT:PSS thin films have angle positions at 2θ = 18° and 26°, which were contributed from PSS at 2θ = 18°. As shown in Figure 2, the XRD pattern of the PSSAS powder revealed an obvious intensity of the angle position at 2θ = 18°, which can be assigned to the (020) planes of the orthorhombic unit cell of PEDOT crystals at 2θ = 26° [21,22,23]. Moreover, all XRD spectra of the PEDOT:PSS thin films that were prepared with various concentrations of PSS were similar to the XRD spectrum of the commercial PEDOT:PSS as shown in Figure 2. In addition, the differences in the intensities of the angle positions at 2θ = 18° and 26° in the XRD spectra of the PEDOT:PSS thin films were due to the differences in the various concentrations of the PSSAS and the solvent treatment with a solvent of pure water mixed with acetone (MeOH or EtOH) in a ratio of 50:50. Thus, in order to further examine this phenomenon, the XRD patterns of the PEDOT:PSS thin films were fitted as shown in Figure 1. The parameters of fitting at 2θ and intensity are summarized in Table 1, with all conductivities and parameters determined as the average value obtained from at least three samples. In addition, the fitted value of 2θ ranged between 17.70° and 18.99° as a fitting for the angle position at 2θ = 18° of the XRD patterns of the PEDOT:PSS thin films. Furthermore, the fitted value of 2θ ranged between 24.25° and 24.82° as a fitting for the angle position at 2θ = 26°. Furthermore, the fitted intensities of the fitting at 2θ = 18° were increased by increasing the concentration of PSSAS but we were interested in the fitted intensities of the fitting at 2θ = 26°. In the untreated cases, the fitted intensities were decreased by increasing the concentration of the PSSAS. It is known that there is an excess amount of PSS in the PEDOT:PSS aqueous solution and PEDOT is surrounded by PSS in order to stabilize its dispersion in water. Moreover, PEDOT chains attach to a PSS chain through Coulombic attraction forces and can have a necklace-like structure [20], which has two conformations of PSS and PEDOT-attached PSS segments. In the case of PEDOT-attached PSS segments, which are screened by the positive charges on the PEDOT in order to reduce the Coulombic repulsions between the PSS anions [24], they have a coil-like structure and the PEDOT-attached PSS segments form blobs to draw the PEDOT away from water. On the other hand, the PSS segments also form a linear structure in order to reduce the Coulombic repulsions between the PSS anions. Furthermore, Coulombic repulsions occur between these PEDOT-attached PSS blobs that have PSS anions in the shell and thus, each blob tries to draw itself away from the others and all blobs are separated as far as possible (Scheme 2). Hence, in the untreated cases, the fitted intensities of the fitting at 2θ = 26° were decreased by increasing the concentration of the PSS, which results in lower crystallinity of the PEDOT chains [19] because each blob tries to draw itself away from the others as far as possible at higher concentrations of the PSS. In contrast, in the solvent-treated cases, the fitted intensities of the fitting at 2θ = 26° followed an order of film B (4.8% PSSAS) > film C (6.2% PSSAS) > film A (2.4% PSSAS). Therefore, the optimal concentration of the PSSAS is between 4.8 and 6.2%.

### 3.2. Electrical Conductivity of the PEDOT:PSS Thin Films

**Figure 3:**
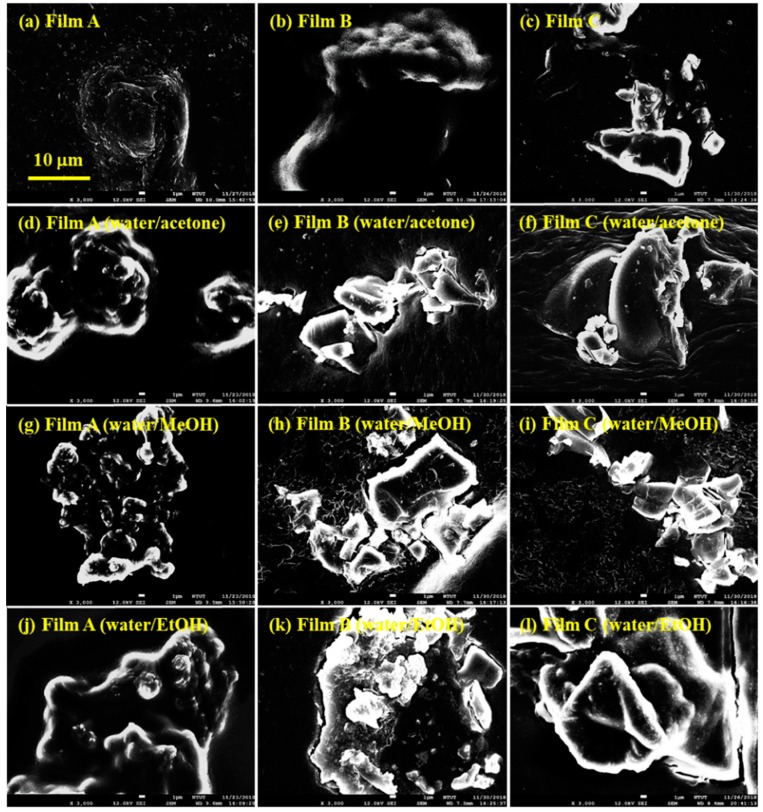
Scanning electron microscope (SEM) images of the PEDOT:PSS thin film A (2.4% PSSAS), B (4.8% PSSAS) and C (6.2% PSSAS) without solvent treatments (**a**–**c**) and with solvent treatments of water/acetone (**d**–**f**), water/methanol (**g**–**i**) and water/ethanol (**j**–**l**). All the images use the same scale bar.

**Figure 4:**
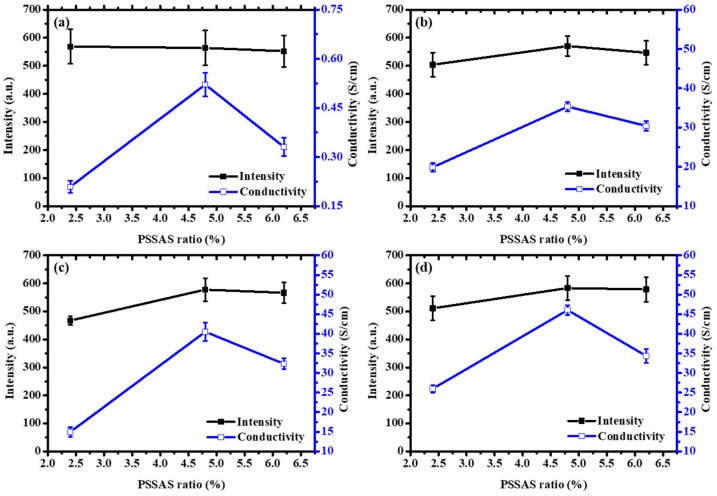
Intensities (black solid square, fitting at 2θ = 26°) and conductivities (blue open square) as functions of PSSAS ratio for PEDOT:PSS thin films with solvent treatments of untreated (**a**), water/acetone (**b**), water/MeOH (**c**) and water/EtOH (**d**).

According to Table 1, the electrical conductivity of solvent-treated films was better than untreated films. These results indicate that the conductivity enhancement in the solvent-treated PEDOT:PSS thin films is different from that in the films that were treated with a polar solvent with a high boiling point, such as EG or DMSO, as the conductivity was greatly enhanced by directly adding a pure organic solvent into the PEDOT:PSS aqueous solution or treating a PEDOT:PSS thin film [24,25,26,27]. In general, organic solvents, such as MeOH and EtOH, can be classified into an OH group and acetone can be classified into a non-OH group. However, there were no obvious differences in the conductivity enhancement between these two groups, which is consistent with our results. On the other hand, high conductivities are consistent with the high dielectric constants of the organic solvents, such as acetone. Moreover, the solvent treatment has effects from solvation [20], with an organic solvent, such as acetone that selectively solvates the PEDOT chains, and water that selectively solvates the PSS chains, which results in synergetic effects from both the organic solvent and water. Moreover, organic solvents mixed with water can reduce the dissociation of PSS into PSS anions and protons [28,29], which subsequently reduces the Coulombic repulsions among the PSS anions in the same polymer chain. In addition, the PSS and PEDOT-attached PSS segments will form a coil-like and a linear (or extended-coil) structure, respectively. Finally, the coil-like PSS segments can facilitate the phase separation of PSS chains from the PEDOT:PSS thin film (Scheme 2). Hence, in our case, acetone (MeOH or EtOH) was mixed with water in a ratio of 50:50 and this mixture was used as a solvent for the treatment of PEDOT:PSS thin films, which resulted in better conductivities than untreated PEDOT:PSS thin films. Figure 3 shows the SEM images of the PEDOT:PSS thin films, which experience significant phase separation with the solvent treatment,. Furthermore, the linear (or extended-coil) structure of the PEDOT-attached PSS segment (Figure 3d–l) is shown compared to the structure of the segment with no treatment (Figure 3a–c). On the other hand, the conductivities of the PEDOT:PSS thin films with the solvent treatment have a trend of film B > film C > film A and the trend is significantly consistent with the trend of the fitted intensities of the fitting at 2θ = 26°. However, the untreated PEDOT:PSS thin films do not show the same trend. Thus, the trend depends on the solvent treatment, as shown in Table 1 and plotted in Figure 4, with intensities at fitting at 2θ = 26° and conductivities as functions of the PSSAS ratio for the PEDOT:PSS thin films. The changing intensities at fitting at 2θ = 26° were attributed to the synergetic effects from both the organic solvent and water. This can reduce the dissociation of PSS into PSS anions and protons, which subsequently reduces the Coulombic repulsions among the PSS anions in the same polymer chain. Thus, the PSS and PEDOT-attached PSS segments will form a coil-like and a linear (or extended-coil) structure, respectively. This may induce the stacking of the linear and planar PEDOT-attached PSS segments, which favors the formation of a crystalline phase [30], so that the XRD intensity increases with an increase in crystallinity. Finally, in our case, the best electrical conductivity of the PEDOT:PSS thin films with the solvent treatment depends on the best XRD intensity of fitting at 2θ = 26° of the film B, which is shown in Figure 4.

## 4. Conclusions

In this work, PEDOT:PSS dispersions with various concentrations of PSSAS (2.4, 4.8, and 6.2%) were synthesized by EDOT in the presence of 200 mL of water. The PEDOT:PSS films were prepared by spin coating so that the PEDOT:PSS dispersions were dropped onto a substrate. The solvent-treated PEDOT:PSS thin films were treated by a solvent of water mixed with acetone (methanol or ethanol) in a ratio of 50:50. In addition, both the organic solvent and water have synergetic effects, which can reduce the dissociation of PSS into PSS anions and protons. This subsequently reduces the Coulombic repulsions among the PSS anions in the same polymer chain. Furthermore, the PSS and PEDOT-attached PSS segments form a coil-like and a linear (or extended-coil) structure, respectively. This may induce the stacking of the linear and planar PEDOT-attached PSS segments, which favors the formation of a crystalline phase. Finally, the best electrical conductivity of the PEDOT:PSS thin films with solvent treatment depends on the best XRD intensity of fitting at 2θ = 26° of the film B (4.8% PSSAS).

## Figures and Tables

**Table 1:** Conductivities and parameters of the fitting XRD spectra of the PEDOT:PSS thin film A (2.4% PSSAS), B (4.8% PSSAS) and C (6.2% PSSAS) with solvent treatments of untreated, water/acetone, water/MeOH and water/EtOH. All conductivities and parameters were determined as the average value of at least three samples.

Solvent Treatment	Films	Fitted 2θ (Degree)	Fitted Intensity (Arbitrary Unit, a.u.)	Conductivity (S/cm)
Untreated	Film A	18.51	527.42	0.21
		24.40	568.51	
	Film B	18.55	539.14	0.52
		24.52	563.85	
	Film C	18.46	544.76	0.33
		24.40	551.90	
Water/acetone	Film A	18.73	458.37	19.89
		24.25	503.96	
	Film B	18.99	543.25	35.33
		24.56	570.61	
	Film C	18.22	554.81	30.43
		24.60	546.81	
Water/MeOH	Film A	18.52	428.14	14.99
		24.28	467.49	
	Film B	18.08	504.42	40.51
		24.65	577.62	
	Film C	18.21	546.55	32.37
		24.82	566.53	
Water/EtOH	Film A	17.70	422.28	25.99
		24.80	511.06	
	Film B	17.89	533.55	46.07
		24.51	583.45	
	Film C	18.09	569.63	34.35
		24.82	578.58	

## Supplementary Materials

The following are available online at http://www.mdpi.com/2073-4360/11/1/134/s1. Table S1: Conductivities of the untreated PEDOT:PSS thin film A (2.4% PSSAS), B (4.8% PSSAS) and C (6.2% PSSAS) with an thermal annealing under air atmosphere at 140 °C for 5 min.
