# Supplementary material for: Characterization of Solvent-Treated PEDOT:PSS Thin Films with Enhanced Conductivities
Source: Polymers (Basel). 2019 Jan 14;11(1):134. doi: 10.3390/polym11010134 (PMC6401880; doi:10.3390/polym11010134)
Supplement: Supplementary file 1 [file polymers-11-00134-s001.pdf]

Characterization of Solvent-Treated PEDOT:PSS Thin Films with Enhanced Conductivities

Syang-Peng Rwei [1,2,*], Yi-Huan Lee [1,2], Jia-Wei Shiu [1,2], Ragu Sasikumar [2] and Uin-Ting Shyr [1,2]

1 Institute of Organic and Polymeric Materials, National Taipei University of Technology, Taipei 10608, Taiwan (R.O.C); yihuanlee@mail.ntut.edu.tw (Y.-H.L.); hahaluckybowen@gmail.com (J.-W.S.); alexilaiho0422@gmail.com (U.-T.S.)

2 Research and Development Center for Smart Textile Technology, National Taipei University of Technology, Taipei 10608, Taiwan (R.O.C); sasi.che2011@gmail.com

* Correspondence: f10714@ntut.edu.tw

Table S1. Conductivities of the untreated PEDOT:PSS thin film A (2.4% PSSAS), B (4.8% PSSAS) and C (6.2% PSSAS) with an thermal annealing under air atmosphere at 140 °C for 5 min.

**Processes**	**Films**	**Conductivity for each sample (S/cm)**	**Average conductivity (S/cm)**
Thermal annealing (110 ℃, 30 min) + without solvent treating + thermal annealing (140 ℃, 5 min)	Film A	0.18<br>0.18<br>0.21	0.19
	Film B	0.52<br>0.54<br>0.53	0.53
	Film C	0.34<br>0.31<br>0.29	0.31
